# Pyrethroid Resistance in *Anopheles gambiae*, in Bomi County, Liberia, Compromises Malaria Vector Control

**DOI:** 10.1371/journal.pone.0044986

**Published:** 2012-09-13

**Authors:** Emmanuel A. Temu, Caroline Maxwell, Godwil Munyekenye, Annabel F. V. Howard, Stephen Munga, Silas W. Avicor, Rodolphe Poupardin, Joel J. Jones, Richard Allan, Immo Kleinschmidt, Hilary Ranson

**Affiliations:** 1 The MENTOR Initiative, Crawley, United Kingdom; 2 Centre for Global Health Research, Kenya Medical Research Institute (KEMRI), Nairobi, Kenya; 3 Vector Group, Liverpool School of Tropical Medicine (LSTM), Liverpool, United Kingdom; 4 National Malaria Control Program, Ministry of Health and Social Welfare, Monrovia, Liberia; 5 Medical Research Council (MRC), Tropical Epidemiology Group, London School of Hygiene and Tropical Medicine (LSHTM), London, United Kingdom; 6 The African Regional Postgraduate Programme in Insect Science (ARPPIS), University of Ghana, Accra, Ghana; 7 The Liberia Africa Indoor Residual Spraying (AIRS) Program, Abt Associates Inc., Bethesda, United States of America; University of Crete, Greece

## Abstract

**Background:**

Long Lasting Insecticidal Nets (LLIN) and Indoor Residual Spraying (IRS) have both proven to be effective malaria vector control strategies in Africa and the new technology of insecticide treated durable wall lining (DL) is being evaluated. Sustaining these interventions at high coverage levels is logistically challenging and, furthermore, the increase in insecticide resistance in African malaria vectors may reduce the efficacy of these chemical based interventions. Monitoring of vector populations and evaluation of the efficacy of insecticide based control approaches should be integral components of malaria control programmes. This study reports on entomological survey conducted in 2011 in Bomi County, Liberia.

**Methods:**

*Anopheles gambiae* larvae were collected from four sites in Bomi, Liberia, and reared in a field insectary. Two to five days old female adult *An gambiae* s.l. were tested using WHO tube (n = 2027) and cone (n = 580) bioassays in houses treated with DL or IRS. A sample of mosquitoes (n = 169) were identified to species/molecular form and screened for the presence of knock down resistance (*kdr)* alleles associated with pyrethroid resistance.

**Results:**

*Anopheles gambiae* s.l tested were resistant to deltamethrin but fully susceptible to bendiocarb and fenithrothion. The corrected mortality of local mosquitoes exposed to houses treated with deltamethrin either via IRS or DL was 12% and 59% respectively, suggesting that resistance may affect the efficacy of these interventions. The presence of pyrethroid resistance was associated with a high frequency of the 1014F *kdr* allele (90.5%) although this mutation alone cannot explain the resistance levels observed.

**Conclusion:**

High prevalence of resistance to deltamethrin in Bomi County may reduce the efficacy of malaria strategies relying on this class of insecticide. The findings highlight the urgent need to expand and sustain monitoring of insecticide resistance in Liberian malaria vectors, evaluate the effectiveness of existing interventions and develop appropriate resistance management strategies.

## Introduction

Malaria remains a major public health problem in Africa and vector control program activities are being substantially scaled up in many malaria endemic countries [1], with some countries considering elimination [2]. Long lasting insecticide treated nets (LLINs) and indoor residual spraying (IRS) form the backbone of these interventions and both have been proven as excellent vector control strategies [3], [4]. Great progress has been made during the past years enabling access to approximately 289 million LLINs in sub-Saharan Africa, enough to cover 76% of the 765 million people at risk of malaria. The number of people protected by IRS in the Africa region increased from 13 million in 2005 to 75 million in 2009, representing approximately 10% of the population at risk [2].

However, the effectiveness of these conventional methods for malaria control faces several challenges. Many of these are logistical including the high cost of scaling up and maintaining coverage. Community compliance is also a challenge in some settings with some malaria endemic countries achieving high LLIN coverage but relatively low usage, particularly during months with lower mosquito densities [5]. Another important challenge is the widespread resistance to pyrethroid insecticides in the major African malaria vectors [6]. LLINs and, to a large extent, IRS are highly dependent on the pyrethroid class of insecticides. Furthermore, durable wall linings (DL), an alternative means of applying long lasting insecticides currently being evaluated, are also presently only available with pyrethoid insecticides [7], [8].

Insecticide resistance is defined as a reduction in the insecticide sensitivity of an insect population and can result in failure of an insecticide to achieve the expected level of control. Insecticide resistance is generally mediated by behavioural, physiological or metabolic factors. The most common and well characterized mechanisms are modification of the insecticide target site and increased metabolism of insecticide. The mechanisms implicated in pyrethroid resistance include metabolic resistance based on elevated levels of cytochrome P450 as well as mutations in the target site, the sodium ion channel, also known as knock down resistance (*kdr)* mutations [9].

Pyrethroid resistance has been increasingly reported from many countries in Africa, especially in West Africa, but there is no published record from Liberia. Most of these reports are based on standard laboratory bioassays [6], but few if any provide insight into the potential operational impact of varying levels of insecticide resistance on the efficacy of the malaria prevention tools and strategies which underline current international efforts to control malaria. Liberia has recently emerged from 14 years of civil war where basic services have been totally disrupted and the National Malaria Control Program (NMCP) is still rebuilding its capacity. However, Liberia was amongst the first countries in Africa to successfully introduce confirmatory diagnosis with rapid malaria tests, and artemisinin combination therapy (ACT) for malaria treatment at national level in 2003. Presidential Malaria Initiative (PMI) malaria operation plan for 2012 indicate approximately 3 million LLINs were distributed in Liberia, with a population of about 3.5 million, between 2005 and 2011 and Liberia malaria indicator survey (LMIS) reported increased national coverage from 6% in 2005 to 47% in 2009 and 50% in 2011 [10]. During the national scale up of LLINs in Liberia, malaria prevalence among children under 5 years old was 32% in 2009 and 28% in 2011 [10]. Despite the high numbers of LLINs distributed, Liberia has been unable to achieve the overall levels of malaria reduction anticipated with this strategy and has been unable to identify the factors that may limit national malaria control success. Routine operational research capacity was destroyed by the war, and is only now being gradually re-established. As a consequence, there has been limited data available on the effectiveness of malaria drugs, insecticides, or malaria prevention tools in general.

This work presents the results from a baseline entomological study conducted in Bomi County in Liberia, in 2011.

## Materials and Methods

### Ethics Statement

Written informed consent was obtained from head of household of the 2 demonstration houses used for entomological testing. Ethical approval for the study was issued by Office of the Institution Review Board, University of Liberia, Monrovia Liberia (FWA00004982 & IOR0004203).

### Study Sites

**Figure 1:**
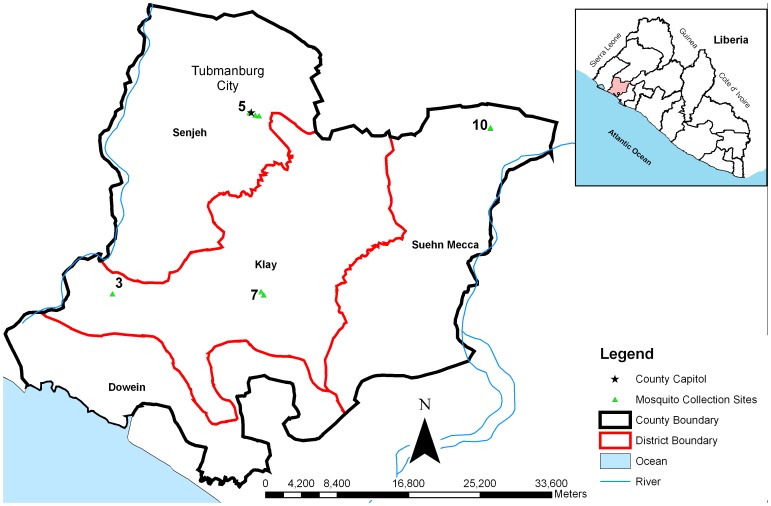
The map of Bomi County showing the location where *Anopheles* larvae were sampled.

The study was conducted in Bomi County situated in the North-western region of Liberia and bordered by Gbarpolu County in the North, Grand Cape Mount County in the West, Montserrado County in the East and the Atlantic Ocean in the South (Figure 1). There are four Administrative Districts (Klay, Dewien, Suehn-Mecca and Senjeh), comprising five Chiefdoms and 18 Clans. The capital city is Tubmanburg located in Senjeh District. Bomi County is generally hilly with a few plains and valleys. The county is endowed with ample water resources (the Po, Wlein, Mahei, Lofa, and St. Paul Rivers) to supply fish and other livelihood options include natural resources such as rubber, timber, diamonds, iron ore, gold, stone and sand, and fertile agricultural land.

The weather in Liberia is generally warm throughout the year and the rainy season begins in May and ends in September. The average annual rainfall of Bomi County is approximately 2030 mm. Malaria transmission is high and perennial, with peak transmission towards the end of the rainy season. *Plasmodium falciparum* is the dominant malaria species in Liberia and the main malaria prevention methods used include the LLINs, IRS and Intermittent Preventive Treatment in Pregnancy (IPTp).

In 2009, a high LLIN ownership (64%) was found in the north western zone of Liberia where Bomi County is located [11]. Net usage for children under 5 years old was 32.7% for any net and 32.2% for LLIN [11]. In 2007-2008, IRS in Liberia was conducted in camps for internally displaced persons and refugees, with a population of approximately 150,000 protected. In 2009, PMI initiated an IRS program in Marshal City (Margibi County) using a pyrethroid insecticide and approximately 22,000 houses were sprayed. Since 2009, IRS activities have increased gradually and in 2011 more than 110,000 houses located in a total of 15 districts in 5 counties were sprayed mainly with pyrethroid insecticides. In 2011, 9% of households in Liberia were sprayed through IRS program [10]. No IRS has ever been conducted in Bomi County.

### Mosquito Larval Collection and Identification of Adult Mosquitoes

Eleven sentinel sites for larval collection were pre-identified in Bomi County. These sites were chosen on the basis of spatial distribution, altitude, proximity to the ocean and whether the sites were in urban or rural areas. Mosquito larval collections were conducted weekly from July to September 2011 [12]. Four sites, each from different administrative districts in Bomi County, yielded sufficient larvae for resistance testing. The larvae were reared to adults at 26±2°C and 70–80% relative humidity, and a cohort of 2 to 5 days old non fed female adults were used for insecticide resistance testing. The four larval collection sites covered a mixture of rural and urban areas with altitude range of 45 to 445 feet above sea level (Figure 1, Table 1).

**Table 1:** The UTM X - Y coordinates for the 4 sites where larvae were collected.

Site	Name	X and Y coordinates
3	Malema Camp	0281721–0738596
	Gissi Camp	0283888–0739927
5	Woman’s centre	0298467–0759538
	UNMIL camp	0297771–0759827
	Garage	0298927–0759467
7	Weamoi	0299144–0738850
	Outside Weamoi	0299466–0738453
10	Outer Norbor	0326054–0758015
	Norbor	0326078–0758087

UTM –The Universal Transverse Mercator geographic coordinate system which uses a 2-dimensional Cartesian coordinate system to give locations on the surface of the earth.

Anopheline larvae were identified morphologically using the Gillies and Coetzee keys [13] to classify them as *An gambiae* s.l. or *An funestus* [14].

### Demonstration Houses for Entomological Testing

Two demonstration houses, one installed with insecticide treated DL, brand name ZeroVector®, and one sprayed with deltamethrin were used for entomological testing. ZeroVector®, manufactured by Vestergaard Frandsen (Switzerland), is a woven shading material made of high-density polyethylene treated with deltamethrin at a concentration of 5 grams/kg [15]. Deltamethrin (K-Othrine) manufactured by Bayer (Germany) was used for IRS with a target surface dose of 250mg per square meter, as per World Health Organization (WHO) specifications [16]. DL was installed on the 14^th^ July 2011 and IRS sprayed on the 2^nd^ September 2011. The lapse in time between the two treatments resulted from late delivery of Deltamethrin (K-Othrine) to Liberia.

### Insecticide Bioassays and Target Site Mutation (*kdr*) Detection

Insecticide susceptibility assays were carried out using 2 to 5 days old *Anopheles* female adults, reared from larvae, following the procedure described by the WHO [17]. Mosquitoes were collected as larvae from four different geographical locations from around Bomi County. These locations can be seen in Figure 1. Mosquitoes from all four locations were used in the WHO insecticide susceptibility tube tests. Insecticide treated test papers were supplied by the Vector Control Research Centre, a WHO Collaborating Centre in Penang, Malaysia and contained the following dosages: deltamethrin (0.05%), bendiocarb (0.1%) and fenithrothion (0.05%). Batches of 15 to 25 mosquitoes per tube were exposed to impregnated papers for one hour. After exposure, mosquitoes were supplied with 10% glucose solution, and mortality was recorded at 24 hours post-exposure. Tests with untreated papers were run as controls. Mortality rate in tested samples was corrected using Abbott’s formula [18] when the mortality rate of control was between 5–20%.

Samples of the same mosquito collections from site 3 Klay District (Figure 1) were used in WHO Cone Bioassays. Batches of 10 to 15 mosquitoes per cone were exposed for 30 minutes to the treated walls in the demonstration houses treated with either IRS or DL. Another subset of mosquitoes was exposed to non-treated walls in separate rooms of the demonstration houses to serve as controls. After exposure, mosquitoes were supplied with glucose solution, and mortality was recorded at 24 hours post-exposure. Mortality rate in tested samples was corrected using the Abbott’s formula [18] when the mortality rate of controls was between 5–20%.

Between 40 and 45 surviving and dead mosquitoes from the cone bioassays using both the DL and IRS treated surfaces were retained for *kdr* analysis. Genomic DNA was extracted using the Livak method [19] and the SINE PCR method [20] was used for species identification and to distinguish *An gambiae* s.s M and S forms. The presence of the 1014F and/or 1014S *kdr* alleles was determined using the Taqman method [21].

## Results

### Insecticide Susceptibility

#### WHO tube bioassay

A total of 2027 *Anopheles* mosquitoes from 4 sites were used in WHO susceptibility tests using bendiocarb, deltamethrin and fenithrothion. These insecticides were selected to represent each of the three classes of chemicals that were either in use, or being considered, for IRS in Liberia (carbamate, pyrethroid and organophosphate respectively). Complete susceptibility to bendiocarb and fenitrothion was obtained in all four sites (Table 2). For deltamethrin, overall mortality observed was between 31.1% to 65.5%, which, according to WHO definitions [17], is indicative of a resistant population.

**Table 2:** Twenty four hours post exposure mortalities rates for *Anopheles gambiae* s.l adults after a one hour exposure to insecticides in WHO tube susceptibility test kits.

Test to C, OP and PY classes of insecticide						Control		
Site	Insecticide	# tested	# dead 24 h	% mortality	Abbott’s correction	# tested	# dead 24 h	% mortality
3	Deltamethrin (PY)	160	58	36.3	**31.1**	160	12	7.5
	Fenitrothion (OP)	122	122	100	100	125	13	10.4
	Bendiocarb (C)	100	100	100	100	100	8	8
5	Deltamethrin (PY)	100	70	70	**65.5**	100	13	13
	Fenitrothion (OP)	100	100	100	100	100	13	13
	Bendiocarb (C)	40	40	100	100	40	2	5
7	Deltamethrin (PY)	115	51	44.4	**36.6**	115	14	12.2
	Fenititrothion (OP)	50	50	100	100	50	2	4
10	Deltamethrin (PY)	100	59	59	**54.9**	100	9	9
	Fenititrothion (OP)	100	100	100	100	100	9	9
	Bendiocarb (C)	25	25	100	100	25	4	16

C – carbamate, OP – organophosphate, PY - pyrethroid.

#### WHO cone bioassay: Identification and detection of *kdr* mutations

Cone bioassays were performed to determine whether the deltamethrin resistance present in *An gambiae* would affect the way the mosquitoes responded to the DL/IRS. A total of 580 *Anopheles* mosquitoes, reared from larvae collected from Malema and Gissi camps (Site 3), was used for cone bioassays in the DL and IRS demonstration houses. Site #3 is located inside the large Sime Darby rubber plantation in Klay district on the west side of Bomi County (Figure 1). An average mortality rate of 63.3% (95% CI: 47.3–73.5) or corrected mortality of 58.7% (95%CI: 42.8–71.6) was obtained after exposure to DL (Table 3). Exposure to IRS house resulted in an average mortality of 19.6% (95%CI: 12.9–24.9) and corrected mortality of 12.0% (95% CI: 8.1–15.9) (Table 4).

**Table 3:** Results of the WHO cone bioassays conducted in the Durable Lining (DL) house using the mosquitoes collected from Site 3 in Klay District.

DL - Deltamethrin test (n = 229)					Control test (n = 58)		
Exposure	# tested	# dead at 24 hrs	% Mortality	Abbott’s correction	# tested	# dead at 24 hrs	% Mortality
**1**	53	33	62.3	**55.9**	14	2	14.2
**2**	59	21	35.6	**30.9**	15	1	6.7
**3**	60	49	81.7	**78.8**	15	2	13.3
**4**	57	42	73.7	**69.3**	14	2	14.3
**Mean**			63.3	**58.7**			12.1

Each test exposure was done on the same day with 4 replicates, each with 10 to 15 mosquitoes.

**Table 4:** Results of the WHO cone bioassays conducted in the Indoor Residual Spraying (IRS) house using the mosquitoe’s collected from Site 3 in Klay District.

IRS - Deltamethrin test (n = 234)					Control (n = 59)		
Exposure	# used	# dead at 24 hrs	% Mortality	Abbott’s correction	# used	# dead at 24 hrs	% Mortality
**1**	59	19	32.2	**27.4**	15	1	6.7
**2**	60	10	16.7	**3.8**	15	2	13.3
**3**	60	9	15.0	**8.5**	14	1	7.1
**4**	55	8	14.6	**8.4**	15	1	6.7
**Mean**			19.6	**12.0**			8.5

Each test exposure was done on the same day with 4 replicates, each with 10 to 15 mosquitoes.

#### Molecular Analysis

A subset of the mosquitoes that were killed or survived the cone bioassays were used for molecular analysis. A total of 169 mosquitoes were identified to species and molecular form. All were *An gambiae* s.s. and 98% of these were the M molecular form, with just three S molecular form mosquitoes identified (all of which survived the cone bioassay).

Taqman assays were used to detect the L1014F and L1014S *kdr* mutations. Out of 169 samples screened for *kdr* mutations, the wild type, homozygote genotype was only detected in dead mosquitoes (around 2.4%). The frequency of the 1014F allele was very high in the M form (90.5%). All three S form *An gambiae* were homozygote for the 1014F *kdr* allele. For both IRS and DL cone bioassays, the frequency of the 1014F *kdr* allele was proportionately higher in the survivors than the dead subset but this difference was only significant in the IRS assay (Table 5). Possessing two copies of the 1014F *kdr* allele increased the chances of surviving the cone bioassay relative to wild type susceptible or heterozygote individuals (OR = 4.2, 95% CI 1.2–16.4 for IRS; OR = 7.4, 95%CI 0.9–341 for DL) (Table 5).

**Table 5:** Results of cone bioassays on deltamethrin Indoor Residual Spraying (IRS) and Durable Lining (DL) surfaces showing the frequency of 1014F *kdr* allele and genotypic recessive odds ratio.

			Genotype					
Surface treated	Mortality Status	Number tested	LL	LF	FF	1014F allele Frequency	Odds ratio^1^ [95% CI]	Fisher’s exact test p-value
IRS	Alive	43	0	5	38	0.94	4.2 [1.2–16.4]	0.01
	Dead	42	2	13	27	0.80		
DL	Alive	40	0	1	39	0.99	7.4 [0.9–341]	0.06
	Dead	44	2	5	37	0.90		

1 Survival of homozygote 1014F *kdr* relative to heterozygotes and *kdr* negatives.

## Discussion

Insecticide bioassay results from Bomi County indicate that the local *An gambiae* has a high prevalence of resistance to the pyrethroid insecticide deltamethrin. This resistance is operationally significant as neither IRS with deltamethrin or DL impregnated with deltamethrin consistently achieved the minimum 80% mortality of mosquitoes needed to meet current WHO standards [22]. Although not tested in this study, the high prevalence of pyrethroid resistance also raises serious concern as to the effectiveness of LLINs in Liberia. All LLINs are currently treated with pyrethroids, and the majority of LLINs distributed to date in Liberia are treated with deltamethrin.

Pyrethroid resistance is due, at least in part, to the presence of the 1014F *kdr* allele. Amino acid substitutions at codon 1014 in the voltage gated sodium channel, the target site of pyrethroids, are a well characterized resistance mechanism in *An gambiae* [23]. This resistance allele is widely distributed throughout Africa and there are indications that pyrethroid-based vector control may be selecting for an increased frequency of this mutation [6]. In western Kenya, dramatic increases in the frequency of the 1014S *kdr* allele in *An gambiae* populations coincided with the scale up of LLIN’s and these same mosquito populations exhibit varying degrees of phenotypic resistance to pyrethroids insecticides [24].

The presence of the 1014F kdr allele is associated with reduced mortality in the cone bioassays, although this is only border-line significant for the DL surface (Table 5). Nevertheless, it is predicted that the 1014F *kdr* allele alone will not compromise control. Although 1014F *kdr* homozygotes had an increased likelihood of surviving exposure (OR = 4.2, 95% CI 1.2–16.4 for IRS; OR = 7.4, 95%CI 0.9–341 for DL) (Table 5) a considerable proportion were killed by the insecticide. Furthermore, despite a *kdr* frequency exceeding 90% in the predominant vector species, approximately 20% of mosquitoes were killed by the IRS and 60% by the DL, indicating that *kdr* alone is not sufficient to compromise control. Presumably, metabolic resistance or other uncharacterised mechanisms are contributing to this resistance phenotype [6].

Once established in a population, resistance can spread very rapidly. Hence a more extensive survey of the insecticide susceptibility status of malaria vectors throughout Liberia is warranted. This information is essential for future planning of malaria vector control efforts in this country. Liberia is currently implementing IRS in 5 of its 15 counties (Montserado, Margibi, Grand Bassa, Bong and Nimba). In 2010, the detection of pyrethroid resistance in two of these counties (Bong and Nimba) prompted a change from pyrethroid to carbamate for IRS (Liberia NMCP, unpublished data).

Encouragingly, bioassay results from Bomi County demonstrated the full susceptibility of *An gambiae* to bendiocarb (carbamate class) and fenitrothion (organophosphates class) insecticides, and both of these insecticides have WHOPES approval for use for IRS. The results from the current study suggest that, if DL is to achieve malaria prevention in this location, it should be impregnated with a different class of insecticide other than pyrethroid.

The selection pressure that is responsible for the high frequency of pyrethroid resistance in Bomi County is unknown. Given the low use of insecticides for agriculture purpose in this region, and the fact that there has never been an IRS program in Bomi County, a plausible explanation is that the resistance to deltamethrin is related to the high coverage of pyrethroid treated LLINs in Liberia [11]. If proved to be the case, this would indicate that universal coverage and continual use of LLINs with same class of insecticide may exert selection pressure on multiple generations of mosquitoes. This is difficult to confirm in Bomi, as no baseline entomological studies were done prior to the start of countrywide scale up of LLIN distribution from 2007. However, this raises an important concern that is worthy of further study in a suitable setting where LLIN universal coverage campaigns have not yet begun.

It is also possible that historical use of insecticide has contributed to the current situation of resistance in Liberia. From 1945 until 1962, Liberia used the organochlorine insecticides (DDT, Dieldrin and BHC) for IRS and as a larvicide for malaria control in the Monrovia city. The program was later extended to the Central Provinces (Kpain), Nimba (Bahn and Saniquellie), the Eastern Province (Tchien) and Maryland County (Harper) [25], but never reached the North-western region of Liberia where Bomi is located. By 1957, *A gambiae* s.l. was reported to be resistant to all of these organochlorines [26]. Since DDT and pyrethroids share the same target site, it is possible that earlier use of this insecticide had helped contribute to the current high prevalence of pyrethroid resistance.

### Conclusion

Pyrethroid resistance raises a concern about the effectiveness of LLINs widely used in Liberia and impose limitations on the insecticides available for IRS. The findings from Bomi County highlight the urgent need to continue monitoring insecticide resistance in Liberian malaria vectors, evaluate the effectiveness of existing interventions in order to provide evidence and develop appropriate resistance management strategies.
